# Adipose-Derived Stem Cells Promote Intussusceptive Lymphangiogenesis by Restricting Dermal Fibrosis in Irradiated Tissue of Mice

**DOI:** 10.3390/ijms21113885

**Published:** 2020-05-29

**Authors:** Ryohei Ogino, Kenji Hayashida, Sho Yamakawa, Eishin Morita

**Affiliations:** 1 Department of Dermatology, Faculty of Medicine, Shimane University, 89-1 Enya-cho, Izumo, Shimane 693-8501, Japan; ryogino@med.shimane-u.ac.jp (R.O.); emorita@med.shimane-u.ac.jp (E.M.); 2 Division of Plastic and Reconstructive Surgery, Faculty of Medicine, Shimane University, 89-1 Enya-cho, Izumo, Shimane 693-8501, Japan; syama8@med.shimane-u.ac.jp

**Keywords:** Adipose-derived stem cell, lymphedema, lymphatic regeneration, x-ray irradiation, fibrosis, intussusceptive lymphangiogenesis

## Abstract

Currently, there is no definitive treatment for lymphatic disorders. Adipose-derived stem cells (ADSCs) have been reported to promote lymphatic regeneration in lymphedema models, but the mechanisms underlying the therapeutic effects remain unclear. Here, we tested the therapeutic effects of ADSC transplantation on lymphedema using a secondary lymphedema mouse model. The model was established in C57BL/6J mice by x-irradiation and surgical removal of the lymphatic system in situ. The number of lymphatic vessels with anti-lymphatic vessel endothelial hyaluronan receptor 1 (LYVE-1) immunoreactivity increased significantly in mice subjected to transplantation of 7.5 × 10^5^ ADSCs. X-irradiation suppressed lymphatic vessel dilation, which ADSC transplantation could mitigate. Proliferative cell nuclear antigen staining showed increased lymphatic endothelial cell (LEC) and extracellular matrix proliferation. Picrosirius red staining revealed normal collagen fiber orientation in the dermal tissue after ADSC transplantation. These therapeutic effects were not related to vascular endothelial growth factor (VEGF)-C expression. Scanning electron microscopy revealed structures similar to the intraluminal pillar during intussusceptive angiogenesis on the inside of dilated lymphatic vessels. We predicted that intussusceptive lymphangiogenesis occurred in lymphedema. Our findings indicate that ADSC transplantation contributes to lymphedema reduction by promoting LEC proliferation, improving fibrosis and dilation capacity of lymphatic vessels, and increasing the number of lymphatic vessels via intussusceptive lymphangiogenesis.

## 1. Introduction

The lymphatic system maintains tissue fluid homeostasis, carries proteins and large molecules, and plays a role in immune response and tumor metastasis [1,2]. Although the incidence of lymphoceles, lymphorrhea, and lymphedema after lymphatic dissection has been declining due to advances in surgical techniques, these iatrogenic disorders still lack definitive treatment and have a strong negative impact on patients’ quality of life [3]. The secondary (acquired) type of lymphedema accounts for the majority of cases worldwide, occurring as a result of trauma, radiotherapy, surgery, infection, or a combination of these [2]. Lymphedema causes fibrosis in surrounding interstitial tissues, which, in turn, exacerbates lymphedema [4]. Therefore, cancer therapy with surgical lymph node dissection and radiotherapy may result in severe impairment of the lymphatic system.

Lymphovenous anastomoses and vascularized lymph node transfer are typical surgical treatments for lymphedema requiring advanced surgical skills, but neither one constitutes definitive treatment [5,6,7,8,9]. Other conservative treatments, such as manual lymph drainage and exercise, also exist. However, conservative treatments do not have strong efficacy because of their poor compliance [10]. There is no curative treatment for lymphedema at present, and radical treatment for lymphedema is required.

Several lymphedema animal models have been developed to elucidate the underlying molecular mechanisms [4,11,12,13,14,15] and to search for regenerative therapies using growth factors or cell transplantation [14,16,17,18,19,20,21,22]. The majority of these models have been prepared in mouse or rat tails, but we wanted a clinically relevant secondary lymphedema model; therefore, we used a chronic secondary lymphedema model in which the left hind limb of mice was x-irradiated followed by surgical division of the superficial lymphatics [12].

Mesenchymal stem cells (MSC), such as bone marrow-derived stem cells (BMSC) and adipose-derived stem cells (ADSCs), are candidates for therapeutic modality for lymphatic disorders because of their multi-differential capacities and paracrine effects. Chen et al. reviewed the cell-based therapy for lymphedema in 11 animal models and seven human studies [10]. Although ADSC therapies have questions about efficacy and safety for human treatment because of lack of study numbers, ADSC is attractive for its abundancy and easy accessibility compared with other MSC sources.

ADSCs are radioresistant, continue to release growth factors in vitro [23], and have the ability of wound healing in irradiated tissue [24,25]. They exert antiwrinkle effects by reducing ultraviolet-B-induced apoptosis, stimulating collagen synthesis of dermal fibrosis [26], and modifying the local microenvironment [27]. Three studies that used ADSC transplantation for the therapy of human lymphedema had been reported [28,29,30]. In these clinical studies, a greater number of lymph nodes and an improvement in the patient-reported outcomes were confirmed after therapies.

Using an x-irradiated secondary lymphedema model mouse, Yoshida et al. reported increased numbers of lymphatic vessels, vascular endothelial growth factor (VEGF)-C and vascular endothelial growth factor receptor (VEGFR)-3 expression, and restoration of lymphatic function in a transplanted ADSC-count-dependent manner [31]. Hayashida et al., using the same model, also reported that a combination of ADSC transplantation with vascularized lymph node transfer resulted in early lymphedema improvement [32]. Additionally, the non-irradiated model confirmed that ADSC transplantation recovered the lymphatic function and reduced lymphedema [17,22,33]. However, similar reports are very limited, and the process of lymphatic vessel regeneration is still unclear.

The aim of this study was to examine the effects of ADSC transplantation on lymphedema caused by x-irradiation and lymphangiogenesis, by analyzing histological changes, fibrosis, and gene expression in regenerative lymphatic vessels. In addition, we predicted the mechanisms underlying lymphatic vessel regeneration.

## 2. Results

### 2.1. Macroscopic Observation of Hind Limb Swelling

**Figure 1:**
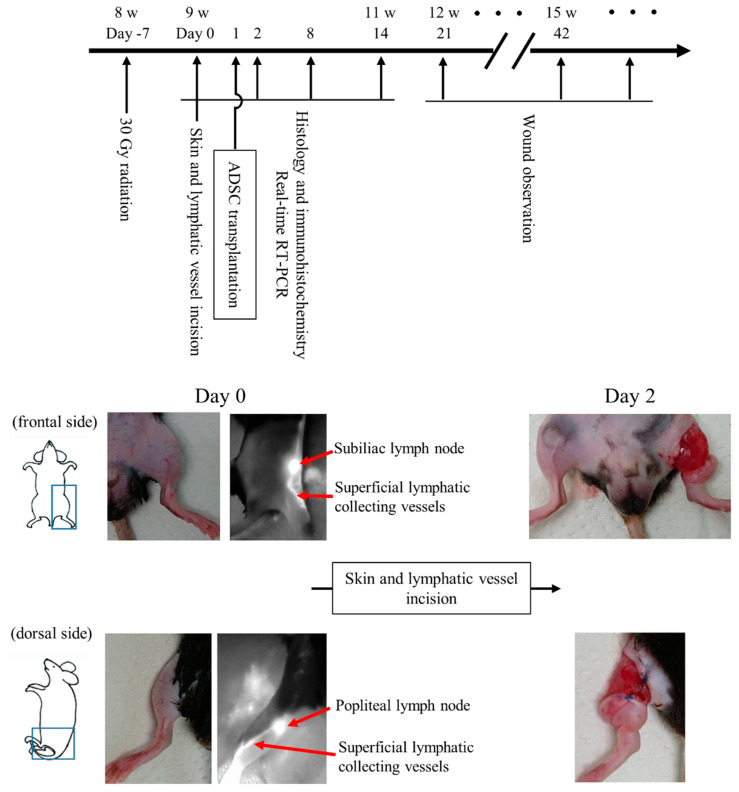
Time course of the experiment and macroscopic image of mouse secondary lymphedema model. Experimental time course and images of skin and lymphatic vessel incisions. The incised lymphatic vessels were identified using a fluorescence near-infrared video camera with intradermal injection of indocyanine green.

The schematic timeline of this study and representative images of the hind limb swelling are shown in Figure 1. All mice showed apparent swelling on the left hind limb within 24 h after surgery. Swelling healed in all mice in the X-ray/ADSC (−/−) group within 21–28 days. In the X-ray/ADSC (+/+) group, swelling improved in 4/6 mice by day 42. In the X-ray/ADSC (+/−) group, improvement time was sparse, and none of the mice improved faster than the mice in the X-ray/ADSC (+/+) group.

### 2.2. The Number and Area of Lymphatic Vessels with LYVE-1 Immunoreactivity

**Figure 2:**
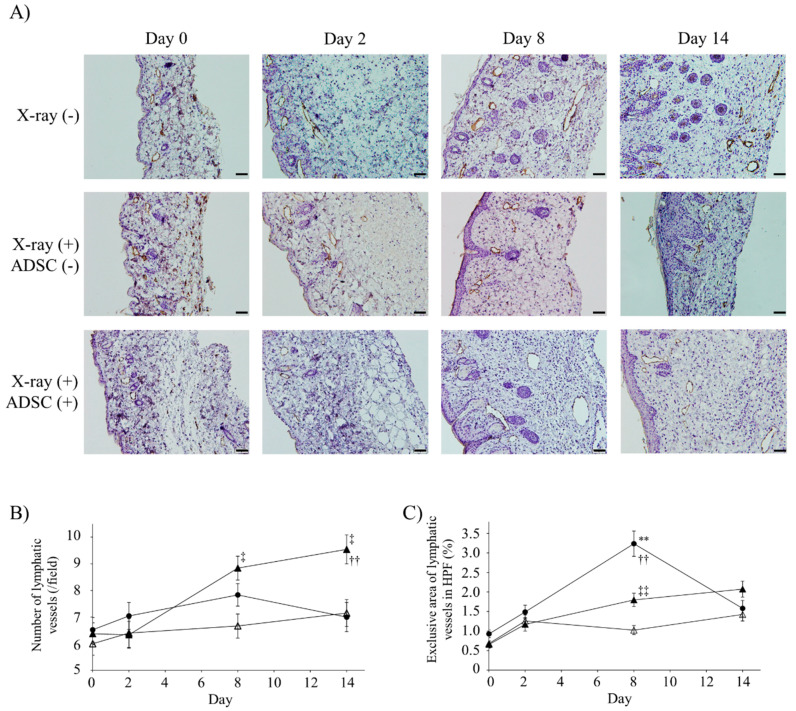
Number and exclusive area of lymphatic vessels with lymphatic vessel endothelial hyaluronan receptor 1 (LYVE-1) immunoreactivity. (**A**) Representative images of immunohistochemistry using anti-LYVE-1 antibody. Scale bars (magnification): 50 µm (200×). (**B**) Number of lymphatic vessels in high power field (HPF) (mean ± SE). (C) Exclusive area of lymphatic vessels in HPF (mean% ± SE). In (B) and (**C**), results of multiple comparisons within the same day groups are indicated. ** *p* < 0.01: significantly different between X-ray/ADSC (−/−) and X-ray/ADSC (+/−). ^††^ *p* < 0.01: significantly different between X-ray/ADSC (−/−) and X-ray/ADSC (+/+). ^‡^ *p* < 0.05, ^‡‡^ *p* < 0.01: significantly different between X-ray/ADSC (+/−) and X-ray/ADSC (+/+). Symbols represent each study group (*n* = 6 mice/group): X-ray/ADSC (−/−) (●), X-ray/ADSC (+/−) (△), X-ray/ADSC (+/+) (▲).

The effects of x-irradiation and ADSC transplantation on lymphatic vessels were evaluated by immunohistochemistry using anti-lymphatic vessel endothelial hyaluronan receptor 1 (LYVE-1) antibody (Figure 2A).

X-irradiation did not affect the number of lymphatic vessels at day 0 (*n* = 6, Figure 2B). The number of lymphatic vessels in the X-ray/ADSC (+/+) group increased significantly compared to that in the X-ray/ADSC (−/−) and X-ray/ADSC (+/−) groups at days 8 and 14, respectively. X-ray/ADSC (+/+) intragroup analysis showed that these numbers increased significantly at days 8 and 14 (mean ± standard error (SE); day 0, day 8, day 14: 6.38 ± 0.41, 8.84 ± 0.45, 9.54 ± 0.55, respectively).

The mean lymphatic vessel area was significantly enlarged in all groups at days 2 and 14 (Table 1). Vessel area in the X-ray/ADSC (−/−) group was further expanded at day 8, compared with that in the two X-ray (+) groups, and percentage of lymphatic vessel area was significantly increased in the X-ray/ADSC (−/−) and X-ray/ADSC (+/+) groups unlike in the X-ray/ADSC (+/−) group at day 8 (Figure 2C).

### 2.3. Analysis of LEC Proliferative Activity

**Figure 3:**
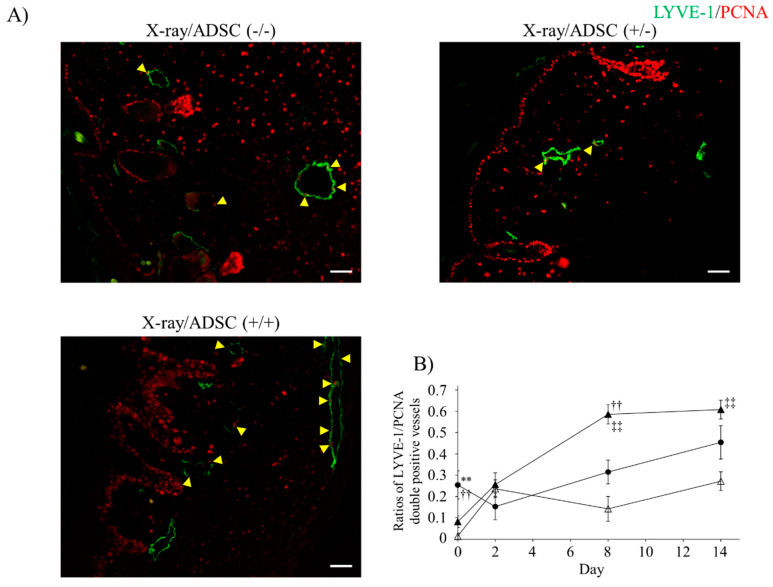
Ratios of proliferative lymphatic vessels with LYVE-1 and proliferating cell nuclear antigen (PCNA) immunoreactivity. (**A**) Representative images of immunofluorescence using anti-LYVE-1 (green) and anti-PCNA (red) antibody at day 8. Arrow heads: LYVE-1 and PCNA double-positive lymphatic endothelial cells. Scale bars (magnification): 50 µm (200×). (**B**) Ratio of proliferative lymphatic vessels (mean ± SE). Results of multiple comparisons within the same day groups are indicated. ** *p* < 0.01: significantly different between X-ray/ADSC (−/−) and X-ray/ADSC (+/−). ^††^ *p* < 0.01: significantly different between X-ray/ADSC (−/−) and X-ray/ADSC (+/+). ^‡‡^ *p* < 0.01: significantly different between X-ray/ADSC (+/−) and X-ray/ADSC (+/+). Symbols represent each study group (*n* = 3–6 mice/group): X-ray/ADSC (−/−) (●), X-ray/ADSC (+/−) (△), X-ray/ADSC (+/+) (▲).

The effects of ADSC transplantation on lymphatic endothelial cell (LEC) proliferative activity were confirmed by immunofluorescence staining using anti-LYVE-1 and anti-proliferating cell nuclear antigen (PCNA) antibodies (Figure 3A). When LYVE-1 positive cells formed a lumen, LYVE-1 and PCNA double-positive cells were considered as the proliferative lymphatic vessel.

Rates of proliferative lymphatic vessels to all lymphatic vessels are shown in Figure 3B and Table 2. On day 0, the proliferative activity of the two X-ray (+) groups was significantly suppressed; lymphatic vessel dissection triggered the increased rates of proliferative lymphatic vessels. In the X-ray/ADSC (+/+) group, the rates significantly increased on day 8 and persisted until day 14, but in the X-ray/ADSC (−/−) group, the increase was not significant.

### 2.4. Evaluation of Fibrosis Using Picro-Sirius Red Staining

**Figure 4:**
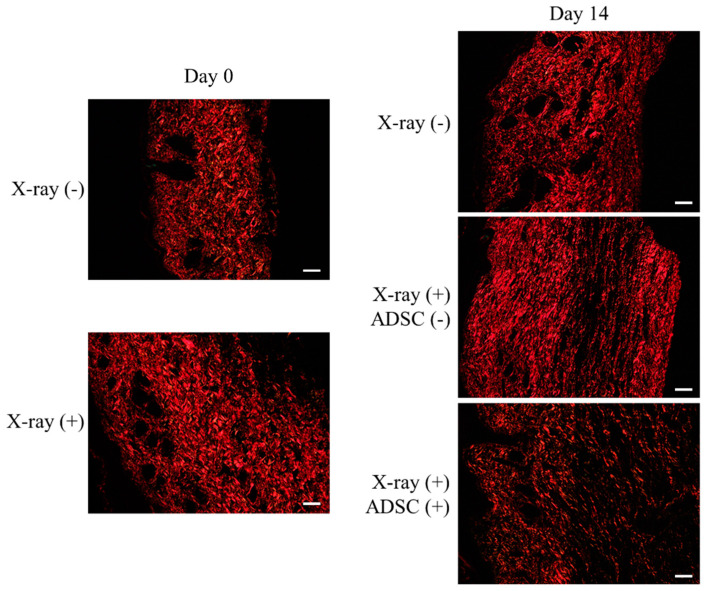
Evaluation of fibrosis using picrosirius red staining. Representative images of picrosirius red staining observed by polarizing microscope. Scale bars (magnification): 50 µm (200×).

Picrosirius red staining was performed to evaluate the severity of skin fibrosis on the left hind limb and the effects of ADSC transplantation (Figure 4, Table 3).

The collagen structure in each group was as follows (Figure 4):

- X-ray/ADSC (−/−) group, day 0: Collagen fibers of type I randomly oriented and type III spread throughout the specimen.
- X-ray/ADSC (−/−) group, day 14: Shorter and still randomly oriented type I collagen fibers; type III collagen fibers only in gaps of type I fibers.
- X-ray (+) groups (X-ray/ADSC (+/−) and X-ray/ADSC (+/+)), day 0: Collagen fibers of type I densely deposited and type III rarely found.
- X-ray/ADSC (+/−) group, day 14: Thicker and longer type I collagen fibers deposited in parallel. Type III collagen fibers only in places of a low density of type I collagen fibers.
- X-ray/ADSC (+/+) group, day 14: Randomly oriented type I collagen fibers with lower density compared to the other two groups. Type III collagen fibers spread throughout the specimen, mainly in gaps of type I collagen fibers.

In the X-ray (+) groups on day 0, the area ratios of red (type I collagen) and green (type III collagen) channels to the whole specimen significantly increased and decreased, respectively. Two weeks after skin and lymphatic vessel incision, both red and green channel ratios in tissues from the X-ray/ADSC (−/−) group significantly decreased. From the two X-ray (+) groups, both channel ratios were further decreased only in tissue from the X-ray/ADSC (+/−) group (Table 3).

Based on the above results, x-irradiation could cause fibrosis, such as changing the orientation of type I and decreasing type III collagen fibers. The therapeutic effect of ADSC transplantation on fibrosis was to recover the orientation of type I collagen fibers from parallel to random and increase the extracellular matrix (ECM), including type III collagen fibers.

### 2.5. Gene Expression Analysis

**Figure 5:**
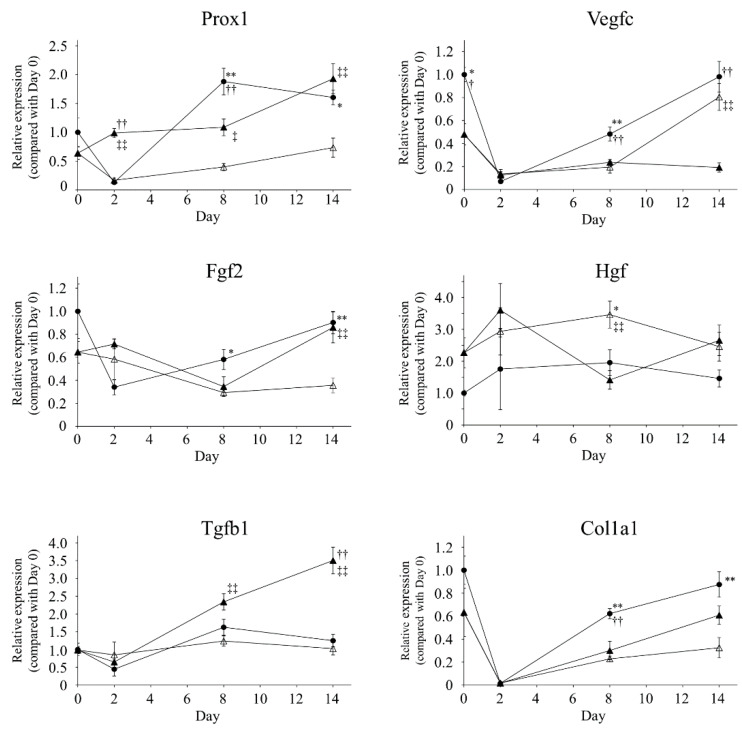
Gene expression analysis. Each gene expression is shown as fold change from the mean value of the X-ray/ADSC (−/−) group at day 0 (mean ± SE). * *p* < 0.05, ** *p* < 0.01: significantly different between X-ray/ADSC (−/−) and X-ray/ADSC (+/−). ^†^ *p* < 0.05, ^††^ *p* < 0.01: significantly different between X-ray/ADSC (−/−) and X-ray/ADSC (+/+). ^‡^ *p* < 0.05, ^‡‡^ *p* < 0.01: significantly different between X-ray/ADSC (+/−) and X-ray/ADSC (+/+). Symbols represent each study group (*n* = 4–6 mice/group): X-ray/ADSC (−/−) (●), X-ray/ADSC (+/−) (△), X-ray/ADSC (+/+) (▲).

mRNA extracted from the skin in edematous regions at days 0, 2, 8, and 14 was used for quantitative RT-PCR. Relative expression values were normalized by the mean value of the X-ray/ADSC (−/−) group at day 0 for each gene. Significant differences among groups are shown in Figure 5.

- X-irradiation did not change *Prox1* expression (day 0, mean ± standard error; X-ray (−), X-ray (+): 1.00 ± 0.25, 0.64 ± 0.11, respectively). In the X-ray/ADSC (+/+) group, *Prox1* expression tended to increase from day 2 and significantly increased at day 14 (day 14: 1.93 ± 0.26, *p* < 0.01 compared with day 0). In the X-ray/ADSC (−/−) group, it increased significantly at day 8 (day 8: 1.88 ± 0.23, *p* < 0.05 compared with day 0).
- X-irradiation significantly suppressed *Vegfc* expression (day 0; X-ray (−), X-ray (+): 1.00 ± 0.06, 0.47 ± 0.09, respectively). Lymphatic incision induced a steep decrease in *Vegfc* expression, and was sustained in the X-ray/ADSC (+/+) group (day14; X-ray/ADSC (−/−), X-ray/ADSC (+/−), X-ray/ADSC (+/+): 0.98 ± 0.13, 0.80 ± 0.12, 0.19 ± 0.14, respectively).
- X-irradiation did not change *Fgf2* expression (day 0; X-ray (−), X-ray (+): 1.00 ± 0.23, 0.64 ± 0.09, respectively). In the X-ray/ADSC (+/+) group, *Fgf2* expression increased significantly at day 14 compared with X-ray/ADSC (+/−) group (X-ray/ADSC (−/−), X-ray/ADSC (+/−), X-ray/ADSC (+/+): 0.90 ± 0.10, 0.35 ± 0.07, 0.86 ± 0.13, respectively).
- Expression of *Hgf* tended to increase, but not significantly, after x-irradiation (day 0; X-ray (−), X-ray (+): 1.00 ± 0.09, 2.27 ± 0.47, respectively). *Hgf* expression did not change significantly during both inter-day or inter-group analysis.
- *Tgfb1* expression did not change by x-irradiation (day 0; X-ray (−), X-ray (+): 1.00 ± 0.19, 0.98 ± 0.10, respectively). Expression of *Tgfb1* mRNA in the X-ray/ADSC (+/+) group significantly increased compared with the other groups from day 8, and increased approximately 3-fold at day 14 (day 14; X-ray/ADSC (−/−), X-ray/ADSC (+/−), X-ray/ADSC (+/+): 1.25 ± 0.17, 1.03 ± 0.18, 3.50 ± 0.37, respectively). In addition, in the X-ray/ADSC (−/−) group, it significantly increased at day 8 (X-ray/ADSC (−/−) day 8: 1.63 ± 0.23, *p* < 0.01 compared with day 0).
- *Col1a1* expression did not change significantly by x-irradiation (day 0; X-ray (−), X-ray (+): 1.00 ± 0.12, 0.63 ± 0.21, respectively). At day 2, expression of *Col1a1* mRNA markedly decreased in all groups (day 2; X-ray/ADSC (−/−), X-ray/ADSC (+/−), X-ray/ADSC (+/+): 0.01 ± 0.01, 0.02 ± 0.01, 0.02 ± 0.00, respectively). When compared with its expression at day 2, that in X-ray/ADSC (−/−) group significantly increased at day 8 (X-ray/ADSC (−/−) day 8: 0.62 ± 0.04, *p* < 0.01) and that in X-ray/ADSC (+/+) group significantly increased at day 14 (X-ray/ADSC (+/+) day 14: 0.61 ± 0.08, *p* < 0.01).

### 2.6. SEM Observation

**Figure 6:**
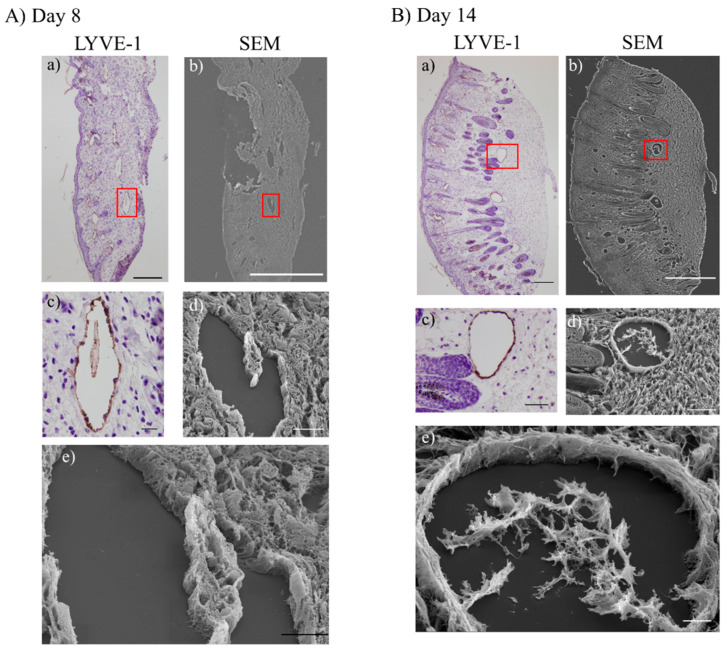
Observation of scanning electron microscopy (SEM) in the X-ray/ADSC (+/+) groups. (**A**,**B**) Overlaid images of serial sections visualized by LYVE-1 immunohistochemistry and SEM observation. Rectangular shapes on (a) and (b) indicate position of high-magnification images (c–e). (**A**): Day 8, (**B**): Day 14. Scale bars (magnification); (**A**-a) 200 µm (40×), (**A**-b) 500 µm (37×), (**A**-c) 20 µm (400×), (**A**-d) 20 µm (900×), (**A**-e) 10 µm (2000×), (**B**-a) 200 µm (40×), (**B**-b) 500 µm (35×), (**B**-c) 50 µm (200×), (**B**-d) 50 µm (300×), (**B**-e) 10 µm (1200×).

To obtain three-dimensional structures of lymphatic regeneration in edematous regions, serial sections were prepared from the mouse left hind limb following surgical lymphatic vessel incision and ADSC transplantation. Lymphatic vessel positions were identified during scanning electron microscopy (SEM) overlaying images of the same sections stained with anti-LYVE-1 antibody (Figure 6).

In the X-ray/ADSC (+/+) groups, dilated lymphatic vessels and protruded LECs toward the lumen observed at days 8 and 14 (i.e., the time the number of lymphatic vessels increased) were considered structures analogous to an intraluminal pillar, the hallmark of intussusceptive angiogenesis. We anticipate that in the lymphedema region, intussusceptive lymphangiogenesis is occurring by restricting dermal fibrosis.

## 3. Discussion

**Figure 7:**
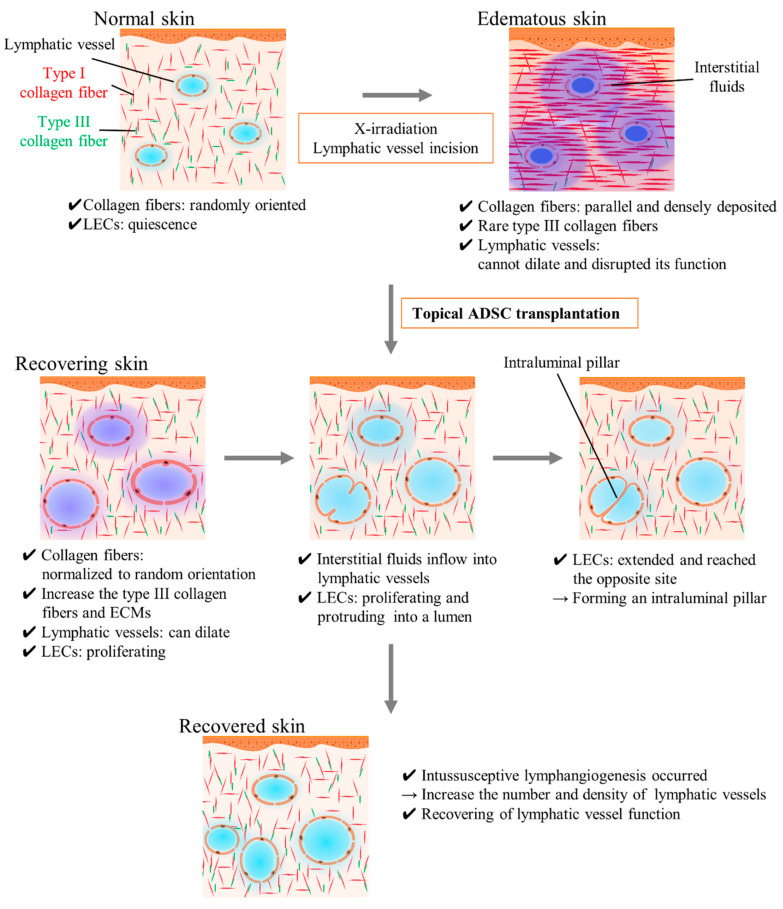
Schematic images of adipose-derived stem cells (ADSC) transplantation effects and intussusceptive lymphangiogenesis. In normal skin, type I collagen fibers are randomly oriented and contribute to appropriate interstitial tissue function. In edematous and recovering skin, type I collagen fibers are parallel deposited and interstitial tissue function is disrupted (e.g., lymphatic vessels cannot dilate and absorb the excessive fluids in the legion). ADSC transplantation improves the fibrosis by recovering the collagen fibers orientation and increasing the number of lymphatic vessels by promoting LEC proliferation and intussusceptive lymphangiogenesis. ADSC, adipose-derived stem cell; ECM, extracellular matrix; LEC, lymphatic endothelial cell.

The analysis of our results indicated that sustained hind limb lymphedema was caused by the inhibition of lymphatic vessel dilation and obstruction of afferent diffusion of interstitial fluid because of the fibrosis of surrounding interstitial tissues and delay of wound healing by x-irradiation. ADSC transplantation mitigated edema by promoting LEC proliferation, increasing the number of lymphatic vessels, accelerating wound healing, and improving dermal fibrosis and lymphatic vessel dilation capacity (Figure 7).

Immunohistochemical staining with anti-LYVE-1 antibody showed that ADSC transplantation increased the number and area of lymphatic vessels compared with the X-ray/ADSC (+/−) group (Figure 2B,C). In an acute lymphedema model, dilated lymphatic vessels were not functional, and interstitial fluid drainage was driven by interstitial forces [4,11,15,21,34]. However, these models do not reflect the severity of fibrosis in a clinical setting because they were prepared without x-irradiation. X-irradiated lymphedema model is considered to cause TGF-β1-mediated tissue fibrosis and interferes with the long-term lymphatic function [35]. In the study, ADSCs improved the severity of interstitial fibrosis; thus, ADSCs may cause drainage of interstitial fluid by interstitial forces and its influx into lymphatic vessels through TGF- β1 pathway.

Immunofluorescence staining with anti-PCNA and anti-LYVE-1-antibodies showed that LEC proliferation was suppressed by 30 Gy x-irradiation a week earlier (Figure 3B). Lymphatic vessel incision and ADSC transplantation stimulated LEC proliferation.

Picrosirius red staining revealed the organization of collagen fibers (Figure 4). The ADSC transplantation group showed a reduction in long and thick type I collagen fibers, a shift from parallel deposition to randomly oriented (normal disposition), and an increased ratio of the green channel, which represented a recovery of type III collagen fibers and ECMs [36,37]. These represent amelioration of fibrosis caused by x-irradiation and lymphatic incision. Fibrosis is one of the major factors that inhibit lymphatic regeneration [4]. ADSC transplantation appears effective against fibrosis because ADSCs and their secreted factors activate dermal fibroblasts and suppress inflammatory cells [26,38]. ADSC transplantation should also be effective against lymphorrhea, promoting lymphatic regeneration and surrounding tissue normalization.

Gene expression analysis revealed ADSC transplantation increased *Prox1*, *Fgf2*, *Tgfb1*, and *Col1a1* mRNA expression compared to those in the X-ray/ADSC (+/−) group at day 14 (Figure 5).

Prox1 is well studied as a master switch determining the LEC fate in developing stages [39,40], but not sufficiently investigated in the adult stage; yet, its expression is considered to indicate switching of the LEC cell cycle from quiescent to proliferative [14]. The increased *Prox1* mRNA expression in the X-ray/ADSC (−/−) group in our study indicate that Prox1 may relate to the lymphatic dilation.

Increased *Fgf2* and *Tgfb* expression was reported in in vitro studies using ADSCs for wound healing [23,41]. Enhancement of type I and type III collagen expression was reported in the ADSC treated group [42], consistent with our picrosirius red staining results. From the viewpoint of lymphatic regeneration and wound healing, we considered that ADSC transplantation was effective in reducing lymphedema by affecting these cytokine profiles.

In contrast, *Vegfc* expression was significantly suppressed in the ADSC transplantation group despite an increase in the number of lymphatic vessels. Although several reports indicate the importance of VEGF-C/VEGFR-3 signaling for lymphangiogenesis [16,43,44,45], prolonged inhibition using the soluble VEGFR-3 decoy receptor did not affect adult lymphatic vessels [46]. Saijo et al. reported VEGF-C and HGF secretion from ADSCs even after γ-irradiation [23]. The mechanisms of lymphatic vasculature network formation, remodeling, and adaptation to physiological and pathological challenges are controlled by an intricate balance of growth factors and biomechanical cues, which transduce signals for the readjustment of gene expression and lymphatic endothelial migration, proliferation, and differentiation [47,48]. ADSCs may relate to the composure of certain ECM proteins [e.g., the collagen- and calcium-binding EGF domains 1 (CCBE1) protein, a disintegrin, and a metalloproteinase with thrombospondin motif 3 (ADAMTS3)], which activate VEGF-C as needed. Further investigations on the efficacy of ADSC treatment for VEGF-C protein quantity and activity are required.

SEM imaging revealed LECs extended toward the inner part of dilated lymphatic vessels in the lymphedema region (Figure 6). This structure resembled the structure of the intraluminal pillar in intussusceptive angiogenesis [49]. Thus, we predicted that intussusceptive lymphangiogenesis by ADSC transplantation occurred in a secondary lymphedema mouse model. Angiogenesis studies described two major modes, sprouting and intussusception [50,51]. The molecular mechanisms underlying intussusception are not fully understood, but several cytokines other than VEGF have been reported. Stromal-derived factor-1 (SDF-1) and its receptor CXC chemokine receptor-4 (CXCR-4), FGF2, inhibition of Notch signaling, shear stress, and nitric oxide are reported to promote intussusceptive angiogenesis [52,53,54,55,56]. Intussusceptive angiogenesis has several advantages: (1) does not require cell proliferation, (2) can rapidly expand an existing capillary network, and (3) can maintain organ function during replication [53]. These advantages are reasonable if intussusceptive lymphangiogenesis occurs in the lymphedema region. Intussusceptive lymphangiogenesis, as a mechanism, is reported in lymphatic malformation/lymphangiomas and sinuses of developing human fetal lymph nodes [57,58]. Díaz-Flores et al. suggested a possible molecular mechanism of intussusceptive lymphangiogenesis in the developing lymph node by which a high abundance of VEGF-C whole lymph node cells, without VEGF-C gradient, results in the nonsprouting engulfment of the lymph node anlage by LECs [57]. Further studies are required to understand the molecular mechanisms and morphology of the lymphatic regeneration at lymphedema status.

In conclusion, ADSC transplantation accelerated LEC proliferation, increased lymphatic vessel numbers, and mitigated fibrosis of the surrounding interstitial tissue. ADSC transplantation efficacy on wound healing is established [24,59,60]. For clinical secondary lymphedema and lymphorrhea, which are thought to be caused by surgical lymph node dissection and radiotherapy, ADSC transplantation is considered to be effective for both lymphatic function recovery and wound healing.

Growth factors secreted by ADSC transplantation (i.e., VEGF, FGF2, TGF-β, HGF, platelet-derived growth factor (PDGF), keratinocyte growth factor (KGF), fibronectin, and collagen I and increased expression of VEGFR-3) promote lymphatic regeneration [24,26,59,61]. Factors associated with lymphangiogenesis and fibrosis include: Tgfb1, suppression of which improves both fibrosis and lymphangiogenesis [18,62]; tumor necrosis factor (TNF)-α and interleukin (IL)-1 [48]; and neutrophils that increase lymphatic vessel density by increasing the number of VEGF-A/VEGFR-2 complexes and releasing VEGF-D [14]. Moreover, factors in the ECM (e.g., CCBE1 and ADAMTS3) required the activation of VEGF-C [47,48] and dipeptidyl peptidase (DPP)-IV expressed on the surface of LEC, interact with the ECM and are involved in LEC migration and tube formation [63]. Thus, the effects of ADSC on lymphedema could be clarified by assessing not only the mRNA level but also the state of these cytokines and complex formations at the protein level.

ADSC transplantation is useful for the prevention and treatment of lymphedema subsequent to irradiation and surgery, improving the fibrosis and recovery of lymphatic function. Further studies are required on the factors that contribute to accelerating lymphatic regeneration effectively.

## 4. Materials and Methods

### 4.1. Animals

C57BL/6J male mice, 5–6-week-old, were purchased from Japan SLC, Inc. (Shizuoka, Japan). The mice were fed a standard laboratory diet and water *ad libitum* for more than 1 week before starting experiments. Experiments involving animals were approved by the Animal Research Committee of Shimane University (Approval No. IZ30-127 on January 23, 2019) and conducted according to the Regulations for Animal Experimentation at Shimane University.

### 4.2. Mouse Secondary Lymphedema Model and Surgical Preparation

A mouse secondary lymphedema model was prepared as previously described [12,32] with minor modification. Briefly, 8-week-old mice (body weight 22.2–26.5 g) were intraperitoneally anesthetized with medetomidine, midazolam, and butorphanol mixed anesthesia and subjected to x-irradiation using MBR-1520R (Hitachi, Ltd., Tokyo, Japan) to the left hind limb at 30 Gy in a single dose 7 days before surgery. After irradiation, mice were intraperitoneally anesthetized and subjected to a circumferential incision in the inguinal region to the muscle layer. Under a microscope, the superficial collecting lymph vessels were cut and cauterized, and the 5-mm wide gap was left open.

### 4.3. Preparation of Adipose-Derived Stem Cells

C57BL/6 mouse ADSCs were purchased from Cyagen (MUBMD-01001, Santa Clara, CA, USA). ADSCs were positive for cell surface antigens CD29, CD44, and Sca-1 and negative for CD31 and CD117. ADSCs were cultured in a 1:1 mixture of Mesenchymal Stem Cell Growth Medium (PT-3001, Lonza, Basel, Switzerland) and Primate ES cell medium (RCHEMD001, REPROCELL Inc., Kanagawa, Japan) supplemented with 50 µg/L of recombinant human basic fibroblast growth factor (REPROCELL). ADSCs were transplanted on mice at passages 2 through 4.

### 4.4. Grouping of the Experimental Animals

Mice prepared for lymphedema were divided into three groups. In the ADSC transplantation groups, 7.5 × 10^5^ ADSCs per mouse were injected with 0.3 mL cell preservative solution (CELLBANKER 2, Takara Bio Inc., Shiga, Japan). Each solution was injected subcutaneously into the distal (to the incised part) limbs and proximal limbs, 24 h after surgery, with a 27-gauge needle. In the no-ADSC transplantation groups, a mock injection of normal saline solution was similarly administered. The three groups consisted of the following:

1. X-ray/ADSC (−/−) group: No-x-irradiated and mock injected group, *n* = 6.
2. X-ray/ADSC (+/−) group: x-irradiated and mock injected group, *n* = 6.
3. X-ray/ADSC (+/+) group: x-irradiated and ADSC transplanted group, *n* = 6.

### 4.5. Histological Examination

Tissue obtained from the distal incision side was washed with 0.1 M phosphate buffer, fixed immediately with 10% formalin/70% methanol solution for 24 h, and embedded in paraffin using Tissue-Tek VIP^®^ 5Jr (Sakura Finetek Japan Co., Ltd., Tokyo, Japan). Embedded specimens were serially sectioned (5 µm).

### 4.6. LYVE-1 Immunoreactivity

Serially sectioned specimens were immersed in xylene for 10 min, then sequentially immersed in 99%, 95%, 90%, 80%, and 70% ethanol for deparaffinization and washed 10 min with distilled water. Antigen retrieval treatment was performed using 0.01 M of citrate buffer (pH 6.0) at 85 °C for 20 min.

For immunohistochemistry, antigen retrieved specimens were immersed in 3% hydrogen peroxide for 15 min, washed with tris(hydroxymethyl)aminomethane-buffered saline (TBS), and incubated with Blocking One Histo (nacalai tesque, Inc., Kyoto, Japan) for 10 min. After washing with TBS-0.1% Tween-20 (TBS-T), they were incubated 1 h at room temperature with rabbit polyclonal anti-mouse LYVE-1 antibody (2 μg/mL; DP3513P, OriGene Technologies Inc., Rockville, MD, USA). The primary antibody was washed out with TBS-T, and specimens were incubated 1 h at room temperature with Donkey Anti-Rabbit IgG H&L (HRP) preadsorbed (1:500 dilution; ab7083, abcam, Cambridge, UK) and visualized with the Peroxidase Stain DAB Kit (nacalai tesque) and hematoxylin. Visualized specimens were mounted with VectaMount^®^ Permanent Mounting Medium (Vector Laboratories Inc., Burlingame, CA, USA) following sequential immersion in 70%, 90%, 95%, 99%, and 100% ethanol and xylene.

Each stained section was scanned at low magnification (40×) to select areas containing the most lymphatic vessels (high power field: HPF). Four HPFs per section were measured at high magnification (200×), and the lymphatic vessel number, mean area, and exclusive area of lymphatic vessels were calculated.

### 4.7. Measurement of LEC Proliferation Activity

For double staining with anti-LYVE-1 and anti-PCNA antibodies, serial sections were processed as in “LYVE-1 immunoreactivity” until antigen retrieval. After incubation with a blocking solution for 10 min and washing with TBS-T, specimens were incubated 1 h at room temperature with anti-LYVE-1 and anti-PCNA antibodies (mouse monoclonal [PC10] to PCNA, ab29, abcam) at 2 μg/mL each. Next, slides were incubated 1 h at room temperature with Donkey Anti-Rabbit IgG H&L (DyLight^®^ 488) preadsorbed (1:500; abcam, ab96919) or Donkey Anti-Mouse IgG H&L (AlexaFluor^®^ 594) preadsorbed (1:200; abcam, ab150112), and mounted with the Vector^®^TrueView^®^ Autofluorescence Quenching Kit (Vector).

Stained sections were visualized using a fluorescent microscope (ECLIPSE 80i, Nikon Corporation, Tokyo, Japan), and scanned at 200× magnification to select the HPF for lymphatic vessels. To identify proliferative LECs, merged images of each channel were created using ImageJ software (version 1.52p, National Institutes of Health, Bethesda, MD, USA). For statistical analysis, four merged images of double-stained regions within each section were selected.

### 4.8. Evaluation of Fibrosis Using Picro-Sirius Red Staining

Collagen fibers in the specimens were visualized using Picrosirius Red Stain Kit (For Collagen) (ScyTek Laboratories, Inc., West Logan, UT, USA). Serial sections were deparaffinized and washed as mentioned in “LYVE-1 immunoreactivity”, covered by picrosirius red solution and incubated for 1 h at room temperature. Specimens were rinsed twice with 0.5% acetic acid and immersed in 70%, 90%, 95%, 99%, and 100% ethanol and xylene.

After mounting as above, specimens were examined using a polarizing microscope (BX-51 with U-POT (polarizer) and U-ANT (analyzer), Olympus Corporation, Tokyo, Japan). To determine the proportions of differently colored collagen fibers, digital images of picrosirius red stained specimens were segmented into two-color threshold bands according to Zerbinati and Calligaro [36] with the slight modification that values were decided as follows (for ImageJ): red channel: hue 0–34, saturation 106–255, brightness 106–186; green channel: hue 35–118, saturation 106–255, brightness 26–186. The entire area of the specimen, which color threshold values were set as default for ImageJ software, and areas of each channel were measured.

### 4.9. Gene Expression Analysis

Tissue obtained from the distal side of the incision was washed with 0.1 M phosphate buffer and immersed in RNA stabilization solution (RNAlater™, QIAGEN, Hilden, Germany). For mRNA extraction, the RNeasy Fibrous Tissue Mini Kit (QIAGEN) was used according to the manufacturer’s instructions. RNA quantity was analyzed using a NanoDrop™ ND-1000 (Thermo Fisher Scientific, Waltham, MA, USA). Reverse transcription was performed using the PrimeScript™ II 1st Strand cDNA Synthesis Kit (Takara Bio Inc.) using 0.5 µg RNA. Real-time RT-PCR was performed using the Thermal Cycler Dice^®^ Real Time System TP860 (Takara Bio Inc.) and Premix Ex Taq™ (Perfect Real Time) (Takara Bio Inc.), according to the manufacturer’s instructions. The following TaqMan^®^ probes, purchased from Thermo Fisher Scientific, were used: prospero homeobox protein 1 (*Prox1*, Mm00435969_m1), vascular endothelial growth factor-c (*Vegfc*, Mm00437310_m1), fibroblast growth factor 2 (*Fgf2*, Mm01285715_m1), hepatocyte growth factor (*Hgf*, Mm01135184_m1), transforming growth factor beta 1 (*Tgfb1*, Mm01178820_m1), collagen type I alpha 1 chain (*Col1a1*, Mm00801666_g1), glyceraldehyde-3-phosphate dehydrogenase (*Gapdh*, Mm99999915_g1). The expression levels of *Prox1*, *Vegfc*, *Fgf2*, *Hgf*, *Tgfb1*, and *Col1a1* were normalized to *Gapdh* expression. A standard curve was prepared using serial dilutions (1-, 10-, 20-, 40-, 80-fold) of control samples and plotted against the cycle numbers obtained at the log-linear phase of the reaction.

### 4.10. Observation Using SEM

For SEM, 15-µm-thick serial sections of paraffin-embedded specimens were prepared and mounted on a MAS-GP type A-coated slide grass (Matsunami Glass Ind., Ltd., Osaka, Japan). In parallel, 5-µm-thick serial sections for LYVE-1 staining were prepared to identify the lymphatic vessel position by SEM. Sections were sequentially immersed in xylene and 99% ethanol for deparaffinization, then transferred in 100% ethanol and tert-butyl alcohol. Slides in frozen tert-butyl alcohol were lyophilized with JFD-320 (JEOL Ltd., Tokyo, Japan), gold ion coated by VX-10A (EIKO ENGINEERING, LTD., Ibaraki, Japan), mounted on an aluminum sample stage using double-sided tape and observed by JSM-6510 (JEOL Ltd.) in secondary electron image mode.

### 4.11. Statistical Analysis

Statistical analyses were performed using RStudio (version 1.1.453, RStudio, Inc., Boston, MA, USA). Statistical significance of differences after x-irradiation at day 0 was determined using Welch’s test, with the *p*-value was set at < 0.05. The overall difference between groups was determined by one-way analysis of variance. Post hoc multiple comparisons were made with Tukey–Kramer test. Values expressed as means ± standard error (SE); values of *p* < 0.05 were considered significant.

## Figures and Tables

**Table 1:** Mean lymphatic vessel areas with lymphatic vessel endothelial hyaluronan receptor 1 (LYVE-1) immunoreactivity (mean ×10^3^ pixel ± SE).

X-ray/ADSC	Day 0	Day 2	Day 8	Day 14
−/−	2.42 ± 0.26	4.25 ± 0.41 **	7.85 ± 0.69 **^,††^	4.77 ± 0.58 **^,‡‡^
+/−	2.21 ± 0.22	4.64 ± 0.55 **	3.51 ± 0.50	4.53 ± 0.90 **
+/+	1.74 ± 0.24	3.49 ± 0.46 *	4.81 ± 0.60 **	5.76 ± 0.74 **

Mean lymphatic vessel area in the high power field (HPF) were calculated following the formula (*n* = 6): [(sum of lymphatic vessel area in HPF)/(number of lymphatic vessels in HPF)]. Lymphatic vessel areas were measured using ImageJ software. Four HPFs per mouse were selected. * *p* < 0.05, ** *p* < 0.01 significantly different from day 0. ^††^ *p* < 0.01 significantly different from day 2. ^‡‡^ *p* < 0.01 significantly different from day 8.

**Table 2:** Ratio of proliferative lymphatic vessels with LYVE-1 and proliferating cell nuclear antigen (PCNA) immunoreactivity (mean ± SE).

X-ray/ADSC	Day 0	Day 2	Day 8	Day 14
−/−	0.25 ± 0.07	0.15 ± 0.06	0.31 ± 0.06	0.45 ± 0.09
+/−	0.01 ± 0.01	0.24 ± 0.04 **	0.14 ± 0.06	0.27 ± 0.04 **
+/+	0.08 ± 0.03	0.26 ± 0.06 *	0.56 ± 0.05 **	0.61 ± 0.04 **

Proliferative lymphatic vessel ratio was calculated following the formula (*n* = 3–6): [(number of LYVE-1 and PCNA double-positive cells forming luminal structure)/(number of all LYVE-1 positive lumen)]. Four HPFs per mouse were selected. * *p* < 0.05, ** *p* < 0.01 significantly different from day 0.

**Table 3:** Percentage of red and green channel area stained by picrosirius red (mean% ± SE).

X-ray	Day 0		X-ray/ADSC	Day 14	
−	Red	21.95 ± 0.63	−/−	Red	19.73 ± 0.83 *
	Green	2.12 ± 0.16		Green	0.90 ± 0.14 *
+	Red	25.42 ± 0.74	+/−	Red	15.06 ± 0.33 **
				Green	0.43 ± 0.06 **
	Green	1.32 ± 0.10	+/+	Red	24.68 ± 1.33
				Green	1.34 ± 0.20

Red and green channel ratio was calculated following the formula (*n* = 3–6): [(area of red or green channel)/(area of whole specimen)]. Each channel area was measured using ImageJ software setting as described in “Evaluation of fibrosis using picrosirius red staining”. The area of the whole specimen was measured using default values of the color threshold automatically set by ImageJ. * *p* < 0.05, ** *p* < 0.01 significantly different from day 0.

## Abbreviations

ADSC	adipose-derived stem cell
BMSC	bone marrow derived stem cell
ECM	extracellular matrix
LEC	lymphatic endothelial cell
LYVE-1	lymphatic vessel hyaluronan receptor 1
MSC	mesenchymal stem cell
PCNA	proliferating cell nuclear antigen
SEM	scanning electron microscopy
TBS	tris(hydroxymethyl)aminomethane-buffered saline
TBS-T	tris(hydroxymethyl)aminomethane-buffered saline containing 0.1% Tween-20
TGF-β	transforming growth factor-beta
VEGF	vascular endothelial growth factor
VEGFR	vascular endothelial growth factor receptor
